# Second-line Treatment of Stage III/IV Non-Small-Cell Lung Cancer (NSCLC) with pemetrexed in routine clinical practice: Evaluation of performance status and health-related quality of life

**DOI:** 10.1186/1471-2407-12-14

**Published:** 2012-01-13

**Authors:** Wolfgang Schuette, Hans Tesch, Hartwig Büttner, Thomas Krause, Victoria Soldatenkova, Clemens Stoffregen

**Affiliations:** 1 Hospital Martha-Maria, Halle-Doelau, Department of Internal Medicine II, Halle, Germany; 2 Hospital Bethanien, Group Practice Oncology, Frankfurt, Germany; 3 Medical Department, Lilly Deutschland GmbH, Bad Homburg, Werner-Reimers Straße 2-4, 61352 Bad Homburg, Germany; 4 Statistics Oncology, Lilly Deutschland GmbH, Bad Homburg, Germany

## Abstract

**Background:**

Second-line treatment of advanced non-small-cell lung cancer (NSCLC) improves overall survival. There is a lack of data regarding the impact on patients' overall health condition. This prospective, non-interventional study evaluated performance status (PS) and health-related quality of life (HR-QoL) during second-line pemetrexed treatment in routine clinical practice.

**Methods:**

Stage III/IV NSCLC patients who initiated second-line pemetrexed (standard vitamin and dexamethasone supplementation) were observed for a maximum of 9 treatment cycles. The primary objective was to evaluate the proportion of patients achieving improvement of Karnofsky Index (KI) of ≥ 10% (absolute) or maintaining KI ≥ 80% after the second treatment cycle ("KI benefit response"). HR-QoL was self-rated using the EuroQoL-5D questionnaire (EQ-5D). Factors potentially associated with KI benefit response were evaluated using logistic regression models.

**Results:**

Of 521 eligible patients (73.5% Stage IV, median age 66.3 yrs, 36.1% ≥ 70 yrs, 62.0% with KI ≥ 80%), 471 (90.4%) completed at least 2 treatment cycles. 58.0% (95%CI 53.6%;62.2%) achieved KI benefit response after the second cycle. Patients with baseline KI ≥ 80%, no Grade 3/4 toxicities during the first 2 cycles, or combination regimen as prior first-line therapy were more likely to achieve a KI benefit response. EQ-5D scores improved over time. Grade 3/4 toxicities were reported in 23.8% of patients (mainly fatigue/asthenia 15.9%, neutropenia 8.7%).

**Conclusions:**

In this large prospective, non-interventional study of second-line pemetrexed treatment in patients with advanced NSCLC, including 36% elderly patients ( ≥ 70 years), physician-rated PS and self-rated HR-QoL were maintained or improved in the majority of patients.

**Trial registration:**

Registered on ClinicalTrials.gov (NCT00540241) on October 4, 2007

## Background

Second-line treatment of advanced non-small-cell lung cancer (NSCLC) improves overall survival (OS), although the survival benefit is still limited (6-8 months) [1-4]. Because the most important purpose of second-line treatment is palliation, maintenance or improvement of a patient's overall health condition is a highly relevant treatment benefit. However, there is a lack of prospective data regarding the impact of second-line treatment on a patient's overall health condition [5]. Patients' overall health condition can be evaluated by looking at their performance status (PS) as rated by a physician, and/or by looking at health-related quality of life (HR-QoL) as rated by the patient themselves. While several clinical studies have shown that patient-rated HR-QoL improves during second-line NSCLC treatment [2,6,7], no study has specifically looked at changes in physician-rated PS. In addition, the few studies that have compared patient-and physician-rated outcomes indicate that physicians tend to rate PS better than the patients themselves [8-11].

We designed a prospective, non-interventional multicenter study in patients whose disease had progressed after first-line chemotherapy for Stage III/IV NSCLC and who were about to start second-line treatment with pemetrexed, to evaluate physician-rated PS and patient-rated HR-QoL during second-line treatment in routine clinical practice. Due to the observational nature of this study, we expected the patient population to be less selected, including a significant proportion of patients with poor PS, as well as elderly patients. For these populations, there is a particular lack of prospective data on tumor response, survival and HR-QoL [12,13]. Elderly patients are of special interest; they are rarely enrolled into clinical trials, but more than two thirds of NSCLC cases are diagnosed in patients aged ≥ 65 years [13]. The primary objective of this study was to evaluate the proportion of patients who achieved an improvement of Karnofsky Index (KI) or maintained a KI ≥ 80% after the second cycle of pemetrexed treatment ("KI benefit response").

## Methods

### Patient population

Adult patients with NSCLC Stage IIIa/b or IV who were about to start second-line treatment with single-agent pemetrexed at the physician's discretion were eligible to participate in this non-interventional study. NSCLC staging was based on the fifth edition of the TNM classification [14] because the study began in 2007.

Pemetrexed has been approved in the USA and Europe for the treatment of patients with advanced NSCLC in the second-line setting since 2004. In 2008, new pemetrexed first-line data revealed that patients with predominantly non-squamous histology have a particular survival benefit [15,16]. Accordingly, the second-line indication for pemetrexed was revised to include patients with advanced NSCLC of predominantly non-squamous histology only. Because our study began in 2007, i.e. before the label change, patients with both squamous and non-squamous histology participated in the study.

Patients were eligible if they had already received one previous cytotoxic chemotherapy regimen; combination with a targeted agent like erlotinib was allowed. However, patients who initially received a targeted agent only and later received cytotoxic chemotherapy were not eligible, because the initial targeted agent treatment would have been regarded as first-line treatment. Previous pemetrexed treatment was not allowed. Physicians routinely involved in the treatment of NSCLC were asked to participate in the study. In- and outpatients were enrolled at 102 hospitals and practices in Germany (100 sites) and Austria (3 sites) between 09/2007 and 08/2009. All patients provided authorization for the use and disclosure of their data collected during this non-interventional study. The study protocol was reviewed by ethical review boards in Germany and Austria (Ethics Committee of the Landesärztekammer Hessen, Frankfurt, Germany; Ethics Committee of the Country of Oberösterreich, Linz, Austria).

### Study design

This prospective, non-interventional phase IV multicenter study (NCT00540241; H3E-SB-B007) evaluated changes in physician-rated PS and patient-rated HR-QoL during second-line treatment with pemetrexed in routine clinical practice. The primary objective was to evaluate the proportion of patients who achieved either an improvement of KI of at least 10% (absolute) or maintained a high KI of at least 80% ("KI benefit response") after the second cycle of second-line pemetrexed treatment, and to identify factors which may be associated with a benefit in KI. The KI benefit response was primarily assessed after the second cycle because physicians routinely evaluate patients' clinical and functional status thoroughly after the second treatment cycle, and most patients can be expected to still be on treatment. Secondary outcomes included patient-rated HR-QoL (European Quality Of Life Five Dimensions questionnaire [EQ-5D]), reasons for treatment discontinuation, evaluation of prior chemotherapy, OS, physician-rated disease control, solicited toxicities, weight loss, and use of solicited disease- or treatment-related concomitant medications.

### Treatment

It was assumed that physicians would administer pemetrexed in accordance with the indication for second-line treatment, as outlined in the current Summary of Product Characteristics (SPC) for the respective countries. According to the SPC, pemetrexed was to be administered every 21 days (1 treatment cycle) at a dose of 500 mg/m^2^, using standard vitamin supplementation and dexamethasone prophylaxis. Any use of further concomitant medication was at the physician's discretion. The duration of pemetrexed treatment was also at the discretion of the physician. In clinical studies, second-line pemetrexed treatment is commonly given over a median of 4 cycles, with few patients exceeding 6 cycles [6]. In this non-interventional study, patients were observed for a maximum of 9 treatment cycles (treatment continued at physician discretion).

### Assessments

The baseline visit was to be scheduled approximately 1 week before administration of the first dose of pemetrexed. On-treatment data were to be collected on day 1 of each treatment cycle, up to the last dose of pemetrexed, but no longer than a maximum of 9 visits. Final data were collected approximately 21 days after the last recorded dose of pemetrexed. Following the last patient visit, all participating physicians were contacted once to collect final follow-up information on patient survival.

Physician-rated KI [17], dose and administration of pemetrexed, body weight, solicited relevant concomitant medications (including vitamin B_12 _supplementation) and survival status were collected at each visit. Overall best response as evaluated by the physician was collected at the final visit. The following solicited toxicities were collected as maximum toxicity grades (none, Grade 1/2, Grade 3/4) at each visit: neutropenia, febrile neutropenia, mucositis, stomatitis, pharyngitis, fatigue, asthenia, nausea, vomiting, diarrhea, and rash/desquamation. An electronic data capture system was used to collect these data. In addition, patients were asked to document folic acid intake in patient diaries and to complete the EQ-5D questionnaire [18] at each visit. The questionnaire comprises 5 dimensions of health (mobility, self-care, usual activities, pain/discomfort, anxiety/depression). Each dimension comprises three levels (some, moderate, extreme problems, rated from 1-3). An overall EQ-5D index was calculated [19], with an index of 1.0 representing full health. In addition, patients evaluated their current health state on a visual analogue scale (EQ-Visual Analogue Scale [VAS]; 0 = worst, 100 = best imaginable health state).

### Statistics

Approximately 580 patients were planned to be observed. Assuming a 5% drop-out rate, this sample size would allow the 95% confidence interval (CI) for the proportion of patients showing a "KI benefit response" to range between 3.6% (for an observed proportion of 4.9%) and 8.4% (for an observed proportion of 50%).

Data analyses were exploratory and performed using SAS software (Statistical Analysis System Release 9.2, SAS Institute). All eligible patients were included in the primary analysis of KI benefit response. Those patients who discontinued prior to the end of the second treatment cycle or who had missing values concerning KI were considered as non-responders in terms of KI benefit response in the primary analysis (worst case scenario). In a second, supportive approach, the analysis was based on all patients who had non-missing data for KI at baseline and after 2 cycles of treatment. For both proportion estimates, 95% CIs based on the F distribution method were provided [20].

Factors potentially associated with achieving a KI benefit response were investigated using logistic regression analysis (univariate and multivariable modeling approaches).

Changes from baseline in EQ-5D index and EQ-VAS scores were explored using two-sided t-tests or sign-tests in case of failed normality assumptions. EQ-5D individual domains, disease control (defined as best response of complete response, partial response or stable disease as evaluated by physician), body weight, concomitant medications and toxicities were analyzed by descriptive statistics. Reasons for discontinuation, type of prior chemotherapy, and solicited toxicities were presented by decreasing frequency. OS was measured from the date of the first pemetrexed dose to the date of death due to any cause. If survival status was unknown at the final follow-up, OS time was censored at the last contact date. The median OS time was estimated using the product limit method [21]. The associated 95% CI was based on the sign test [22]. Follow-up time was quantified using the "reversed" Kaplan-Meier method [23].

## Results

### Patient disposition

Of 542 NSCLC patients with any documentation available, 521 (96.1%) patients were eligible for analysis. One patient was excluded because he had received no previous chemotherapy, 19 patients because they had received more than 1 chemotherapy regimen prior to study entry. One patient had been set up in the electronic data capture system before any treatment decision was made and was excluded because there was no further documentation and no plan to start second-line pemetrexed treatment. Of the 521 eligible patients with baseline data, 516 (99.0%) received at least one dose of pemetrexed (2 patients died before the first dose, 3 patients were lost to follow-up or had missing data). 471 patients (90.4%) completed at least 2 cycles of pemetrexed treatment. 254 patients (48.8%) completed at least 6, and 110 (21.1%) at least 9 treatment cycles. 25 patients (4.8%) continued pemetrexed after the end of the observational period. A median of 5 treatment cycles (range 1 to 9) were documented.

Of all patients treated (N = 516), 28.9% completed the treatment schedule as planned by the physician. The most frequent reasons for early discontinuation of pemetrexed were disease progression (27.7%), followed by death (14.3%), patient decision (14.1%), toxicity (4.5%), other reasons (3.9%) and loss to follow-up (1.2%).

### Patient characteristics

Table 1 presents the baseline characteristics for all eligible patients (N = 521). The majority of all patients (73.5%) had Stage IV disease, 37.8% of patients had a poor baseline KI of < 80%. Patients overall had a median age of 66.3 years, and 36.1% of patients were ≥ 70 years old. The elderly patients were similar to the overall population in terms of baseline KI and histology (Table 2).

**Table 1:** Baseline characteristics, all patients

	All patients (N = 521)
**Age**	

Years, median (range)	66.3 (39-86)

≥ 70 years, n (%)	188 (36.1)

Gender, n (%) male	363 (69.7)

Origin, n (%) Caucasian	514 (98.7)

Weight, kg [mean (SD)]	74.9 (14.45)

**Smoking status, n (%)**	

Current smoker	139 (26.7)

Ex-smoker	243 (46.6)

Never smoked	138 (26.5)

Missing data	1 ( 0.2)

**Histology, n (%)**	

Non-squamous cell carcinoma	444 (85.2)

Squamous cell carcinoma ^a^	77 (14.8)

**Disease stage at study entry, n (%)**	

Stage IIIa	35 ( 6.7)

Stage IIIb	103 (19.8)

Stage IV	383 (73.5)

	**All patients (N = 521)**

**Karnofsky Index (KI), n (%)**	

KI ≥ 80%	323 (62.0)

KI 70%	134 (25.7)

KI 60%	45 ( 8.6)

KI 50%	18 ( 3.5)

Missing data	1 ( 0.2)

**Type of previous first-line chemotherapy**	

Platinum-based combinations, n (%)	450 (86.4)

Platinum-free combinations, n (%)	12 ( 2.3)

Single-agent, n (%)	59 (11.3)

Most frequent agents used (at least 10% of patients), n (%)^b^	

Carboplatin	327 (62.8)

Cisplatin	135 (25.9)

Vinorelbine	162 (31.1)

Gemcitabine	159 (30.5)

Paclitaxel	131 (25.1)

Duration of previous first-line chemotherapy, months [median (range)]	3.5 (0-32)

Time elapsed since previous first-line chemotherapy, months [median (range)]	3.9 (0-74)

**Patients [n (%)] who started pemetrexed**	

Immediately after end of first-line treatment^c^	82 (15.7)

Within 3 months after end of first-line treatment^d^	132 (25.3)

More than 3 months after end of first-line treatment^e^	302 (58.0)

Missing data	5 (1.0)

N = total number of patients, n = number of patients, *SD *standard deviation

^a) ^The study started in 2007, before the label change in April 2008 which restricted the use of pemetrexed to patients with non-squamous cell carcinoma

^b) ^Multiple nominations possible

^c) ^First dose of pemetrexed given ≤ 28 days after the last dose of first-line therapy

^d) ^First dose of pemetrexed dose given > 28 days but ≤ 3 months after the last dose of first-lintherapy

^e) ^First dose of pemetrexed dose given > 3 months after the last dose of first-line therapy

**Table 2:** Disease characteristics by age group

	Age < 70 years (N = 333) n (%)	Age ≥ 70 years (N = 188) n (%)	All patients (N = 521) n (%)
**Histology**			

Non-squamous cell carcinoma	283 (85.0)	161 (85.6)	444 (85.2)

Squamous cell carcinoma^a^	50 (15.0)	27 (14.4)	77 (14.8)

**Disease stage at study entry**			

Stage IIIa	18 ( 5.4)	17 ( 9.0)	35 ( 6.7)

Stage IIIb	65 (19.5)	38 (20.2)	103 (19.8)

Stage IV	250 (74.1)	133 (70.7)	383 (73.5)

**Karnofsky Index (KI)**			

KI ≥ 80%	207 (62.2)	116 (61.7)	323 (62.0)

KI 70%	89 (26.7)	45 (23.9)	134 (25.7)

KI 60%	27 ( 8.1)	18 ( 9.6)	45 ( 8.6)

KI 50%	10 ( 3.0)	8 ( 4.3)	18 ( 3.5)

Missing data	0 ( 0.0)	1 ( 0.5)	1 ( 0.2)

N = total number of patients, n = number of patients

^a) ^The study started in 2007, before the label change in April 2008 which restricted the use of pemetrexed to patients with non-squamous cell carcinoma

Regarding previous first-line chemotherapy, 86.4% of all patients had received platinum-based combinations (Table 1). These were carboplatin-based in approximately two thirds of the patients (overall use: carboplatin 68.2%, cisplatin 25.9% of patients). Median duration of prior chemotherapy was 3.5 months. The majority of patients (58.0%) had stopped prior chemotherapy (last dose) more than 3 months before the start of this study.

### Dose administration

The median initial pemetrexed dose was 500 mg/m^2 ^(range 330 to 500 mg/m^2^). Overall, 232 patients (49.3%) had at least one dose delay, but only 33 patients (7.0%) had a dose delay due to toxicity. Doses were mainly delayed due to scheduling conflicts (35.0%). At least one dose reduction during the study was documented for 25 (4.8%) patients. Of these patients, 10 (40.0%) received a dose ≤ 75% of the previous dose and 1 patient (4%) received a dose of ≤ 50% of the previous dose.

### Physician-rated PS (KI)

**Figure 1:**
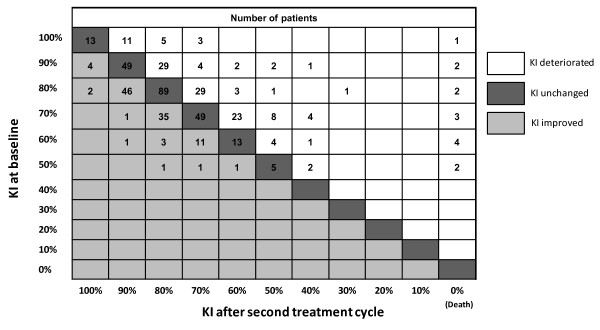
**Shift table presenting the number of patients by baseline KI and KI after second treatment cycle with pemetrexed**. KI = Karnofsky Index

In this study, 58.0% of patients (302 of 521 patients; 95% CI 53.6%; 62.2%) achieved a "KI benefit response" after the second treatment cycle; KI improvement by at least 10% was achieved in 20.3% of patients and 37.6% maintained a high KI of at least 80% (Table 3). Of note, 20.8% of patients with a poor baseline KI < 80% had improved to a KI of at least 80% after the second treatment cycle (41of 197 patients) (Figure 1). 15.8% of patients with a high baseline KI (51 of 323 patients) deteriorated to a KI below 80% after the second cycle, 24 patients had missing data after the second cycle. If patients with missing data were excluded from the analysis, the KI benefit response rate was 64.1% (95% CI: 59.6%; 68.5%). After the sixth treatment cycle, 74.4% of 254 patients still on treatment showed KI benefit response (95% CI 68.6%; 79.7%). Median KI remained unchanged at 80% throughout the study.

**Table 3:** KI benefit response after the second treatment cycle (worst case scenario)

	KI benefit response ^a ^All patients (N = 521) n (%) of patients [95% CI]
**Responders overall**	**302 (58.0) [53.6; 62.2]**

0% improvement of KI, but maintained ≥ 80%	151 (29.0)

10% improvement of KI	97 (18.6)

20% improvement of KI	7 ( 1.3)

30% improvement of KI	2 ( 0.4)

Worsening of KI, but still maintained ≥ 80%	45 ( 8.6)

**Non-responders, overall**	**219 (42.0) [37.8; 46.4]**

0% worsening of KI, but maintained < 80%	67 (12.9)

Deterioration of KI, developed KI < 80%	102 (19.6)

Missing data ^b^	50 ( 9.6)

*CI *confidence interval, *KI *Karnofsky Index, N = total number of patients, n = number of patients

^a) ^Defined as improvement in KI of at least 10% (absolute) or maintenance/achievement of an absolute KI of at least 80%

^b) ^Patients who discontinued prior to the end of the second treatment cycle or who had missing KI data were considered as non-responders (worst case scenario)

**Figure 2:**
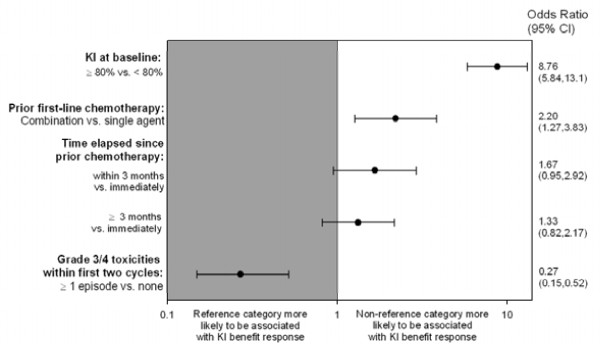
**Odds ratios for patients' characteristics potentially associated with KI benefit response after the second treatment cycle**. CI = confidence interval, KI = Karnofsky Index. Odds ratios derived from univariate regression analyses, including all eligible patients (N = 521). Reference categories: KI at baseline = < 80%, prior first-line chemotherapy = single agent, time elapsed since prior chemotherapy = immediately, Grade 3/4 toxicities = none. Time elapsed since prior chemotherapy: Immediately = first dose of pemetrexed given ≤ 28 days after the last dose of first-line therapy. Within 3 months = first dose of pemetrexed given > 28 days but ≤ 3 months after the last dose of first-line therapy. Later than 3 months: first dose of pemetrexed given > 3 months after the last dose of first-line therapy

Figure 2 summarizes the analysis of potential factors associated with the KI benefit response after the second treatment cycle (univariate logistic regression analyses).

There was a significant advantage with respect to achieving KI benefit response after the second treatment cycle for patients who had a KI of ≥ 80% at baseline (*p *< 0.001) and for patients who had received first-line combination treatment as when compared with those who had received single-agent treatment (*p *= 0.005). Time elapsed since first-line chemotherapy had no significant impact on KI benefit response. Patients who had experienced Grade 3/4 toxicities during the first 2 treatment cycles had significantly less chance of achieving KI benefit response (*p *< 0.001). These results of the univariate models were supported by multivariable modeling approaches which gave similar results (data not shown).

### Patient-rated HR-QoL (EQ-5D)

**Figure 3:**
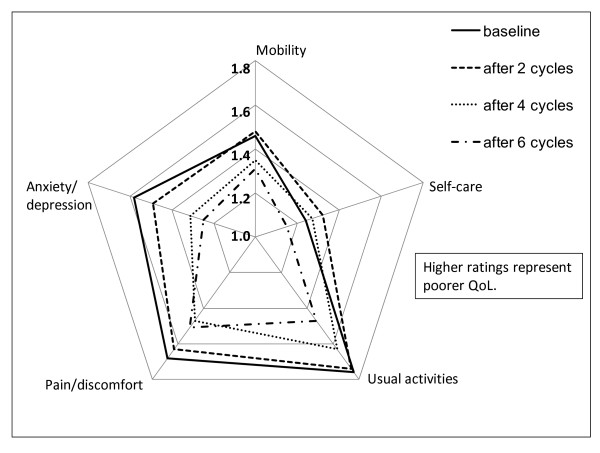
**EQ-5D health status profiles: mean ratings for each of the 5 dimensions of the EQ- 5D questionnaire over time**. On the EQ-5D questionnaire, the patient rates each of the 5 dimensions (mobility, self care, usual activities, pain/discomfort and anxiety/depression) as associated with "some problems" (= 1), moderate problems (= 2), extreme problems (= 3). EQ- 5D = European Quality Of Life Five Dimensions questionnaire, QoL = Quality Of Life, n = number of patients

Approximately half of the patients returned completed EQ-5D questionnaires and therefore were evaluable for HR-QoL. Patients' mean EQ-5D index at baseline was 0.66 (standard deviation [SD] 0.256; N = 231 patients with data available). A small, statistically significant improvement of this score was noted after the second treatment cycle, that is after approximately 6 weeks of treatment (mean increase 0.02, SD 0.214; N = 190 patients; *p *= 0.003). For those patients remaining on study, the EQ-5D index continued to improve up to treatment cycle 6 (mean increase 0.11, SD 0.228; N = 61 patients; *p *< 0.001). Figure 3 presents patients' mean EQ-5D ratings for the 5 individual EQ-5D domains at baseline and after the second, fourth and sixth treatment cycle. For the pain/discomfort and anxiety/depression dimensions, improvements had started after the second treatment cycle. Improvements in mobility and usual activities became visible after the fourth cycle only. Patient self-care worsened initially after the second cycle, but then improved up to treatment cycle 6.

Patients' self-ratings of overall health status on the EQ-VAS gave consistent results. The mean baseline EQ-VAS score was 59.3 (SD 17.80; N = 225 patients). There was a small, statistically significant improvement after the second cycle (mean increase 3.3, SD 12.58; N = 182 patients; *p <*0.001). EQ-VAS ratings continued to improve significantly up to treatment cycle 6 (mean increase 12.8, SD 17.62; N = 60 patients; *p <*0.001).

### Disease control and overall survival

The overall disease control rate, with disease control defined as physician-evaluated best response of complete or partial response or stable disease, was 60.3% (314 patients; 95% CI 55.9%; 64.5%). After a median follow-up time of 13.8 months, the median survival time was 11.1 months (95% CI 9.5 months; 12.2 months). The 12-month survival rate was 45.8% (95% CI 40.7%; 50.7%), 25.6% of patients were still at risk (i.e., still under follow-up).

### Solicited toxicities, weight control and blood transfusions

The analysis of toxicities which were collected by solicited questioning included 516 patients who received at least one dose of pemetrexed. Overall, any Grade 3/4 toxicities were reported for 23.8% of patients. Two patients (0.4%) died due to toxicity during the first treatment cycle. Grade 3/4 toxicities reported for these patients included febrile neutropenia for one patient (male, 65 years, KI 90%), and fatigue/asthenia, mucositis, neutropenia, and stomatitis/pharyngitis for the second patient (male, 69 years, KI 70%). Grade 3/4 toxicities occurred most frequently during the initial cycle (11.0% of patients), less frequently during cycles 2 to 4 (8.5%, 9.0%, and 9.3% of patients, respectively), and then decreased sharply after the fourth cycle (< 5% for all cycles after cycle 4).

Table 4 summarizes the frequency of these solicited toxicities by maximum toxicity grade as evaluated by the physicians, for all patients, by baseline KI status and by age group. Grade 3/4 toxicities reported in more than 2% of patients were fatigue/asthenia (15.9%), neutropenia (8.7%) and nausea (3.1%). Grade 3/4 toxicity rates were twice as high in patients with poor baseline KI (< 80%) when compared to patients with a baseline KI of ≥ 80% (34.7% vs. 17.4%; see Table 4). There was no evidence that Grade 3/4 toxicity rates were higher in elderly than in younger patients ( ≥ 70 vs. < 70 years).

**Table 4:** Solicited Grade 3/4 toxicities (physician evaluation) by baseline KI and age group

		n (%) of patients with maximum Grade 3/4 toxicity			
	
Toxicity	All patients treated (N = 516)	Baseline KI ≥ 80% (N = 322)	Baseline KI < 80% (N = 193)	Age < 70 years (N = 329)	Age ≥ 70 years (N = 187)
Any toxicity	123 (23.8)	56 (17.4)	67 (34.7)	87 (26.4)	36 ( 19.3)

Fatigue/asthenia	82 (15.9)	32 ( 9.9)	50 (25.9)	54 (16.4)	28 ( 15.0)

Neutropenia	45 ( 8.7)	21 ( 6.5)	24 (12.4)	30 ( 9.1)	15 ( 8.0)

Nausea	16 ( 3.1)	6 ( 1.9)	10 ( 5.2)	10 ( 3.0)	6 ( 3.2)

Febrile neutropenia	10 ( 1.9)	3 ( 0.9)	7 ( 3.6)	6 ( 1.8)	4 ( 2.1)

Rash/desquamation	7 ( 1.4)	6 ( 1.9)	1 ( 0.5)	4 ( 1.2)	3 ( 1.6)

Stomatitis/pharyngitis	7 ( 1.4)	3 ( 0.9)	4 ( 2.1)	3 ( 0.9)	4 ( 2.1)

Mucositis	5 ( 1.0)	1 ( 0.3)	4 ( 2.1)	3 ( 0.9)	2 ( 1.1)

Vomiting	5 ( 1.0)	2 ( 0.6)	3 ( 1.6)	4 ( 1.2)	1 ( 0.5)

Diarrhea	2 ( 0.4)	1 ( 0.3)	1 ( 0.5)	2 ( 0.6)	0 ( 0.0)

*KI *Karnofsky Index, N = total number of patients, n = number of patients

Analysis of body weight showed that weight loss was rare. Only 5.4% of all patients lost ≥ 10% of body weight during the period of documentation. Red blood cell (RBC) transfusions were required by 24.2% of patients at some time during the study; 9.2% of patients received a RBC transfusion at baseline, 3.9% and 5.5% of patients received RBCs at the time of the first and second pemetrexed treatment, respectively (Table 5). Platelet transfusions were required by 3.5% of patients, colony-stimulating factors were administered to 11.1% of patients. 95.2% of patients received dexamethasone prophylaxis (or other steroids) at some time during the study, 97.3% of patients received at least one dose of vitamin B_12_. Further, 83.5% of patients received antiemetics, 74.3% received analgesics, and 12.7% received antidiarrheals. Intake of at least one dose of folic acid was documented by 98.3% out of 292 patients with diary information available [24].

**Table 5:** Disease- or treatment related concomitant medication as reported by physicians

Cycle	N	Analgesics n (%)	Antiemetics n (%)	Antidiarrheals n (%)	Dexa or similar steroids n (%)	CSF n (%)	Other medications n (%)	RBC transfusions n (%)	Platelet transfusions n (%)
Overall	521	387 (74.3)	435 (83.5)	66 (12.7)	496 (95.2)	58 (11.1)	292 (56.0)	126 (24.2)	18 ( 3.5)

Baseline	521	270 (51.8)	182 (34.9)	6 ( 1.2)	244 (46.8)	12 ( 2.3)	187 (35.9)	48 ( 9.2)	5 ( 1.0)

Cycle 1	516	265 (51.4)	270 (52.3)	7 ( 1.4)	452 (87.6)	5 ( 1.0)	196 (38.0)	20 ( 3.9)	2 ( 0.4)

Cycle 2	471	242 (51.4)	288 (61.1)	20 ( 4.2)	430 (91.3)	14 ( 3.0)	201 (42.7)	26 ( 5.5)	0 ( 0.0)

Cycle 3	421	226 (53.7)	260 (61.8)	16 ( 3.8)	391 (92.9)	14 ( 3.3)	186 (44.2)	29 ( 6.9)	4 ( 1.0)

Cycle 4	344	178 (51.7)	215 (62.5)	15 ( 4.4)	327 (95.1)	17 ( 4.9)	155 (45.1)	30 ( 8.7)	3 ( 0.9)

Cycle 5	278	142 (51.1)	179 (64.4)	8 ( 2.9)	268 (96.4)	11 ( 4.0)	137 (49.3)	14 ( 5.0)	1 ( 0.4)

Cycle 6	254	129 (50.8)	168 (66.1)	7 ( 2.8)	244 (96.1)	9 ( 3.5)	125 (49.2)	15 ( 5.9)	0 ( 0.0)

Cycle 7	150	93 (62.0)	87 (58.0)	4 ( 2.7)	143 (95.3)	4 ( 2.7)	96 (64.0)	7 ( 4.7)	0 ( 0.0)

Cycle 8	135	88 (65.2)	64 (47.4)	5 ( 3.7)	131 (97.0)	4 ( 3.0)	87 (64.4)	6 ( 4.4)	1 ( 0.7)

Cycle 9	110	75 (68.2)	52 (47.3)	2 ( 1.8)	105 (95.5)	1 ( 0.9)	77 (70.0)	4 ( 3.6)	0 (0.0)

Final	516	289 (56.0)	253 (49.0)	21 ( 4.1)	317 (61.4)	25 ( 4.8)	203 (39.3)	60 (11.6)	8 ( 1.6)

*CSF *colony stimulating factors, *Dexa *dexamethasone, N = number of patients per cycle, *RBC *red blood cells

## Discussion

Second-line treatment improves survival and is increasingly used in patients with advanced NSCLC [2,5,25]. Prospective data evaluating patients' overall health condition during second-line treatment as reflected by PS or HR-QoL are still rare, in particular for elderly patients or patients with poor PS as frequently treated in routine clinical practice. Patients older than 70 years are of special interest; even their first-line treatment is under continuous debate [25,26] because multimorbidity and a higher incidence of toxicities are assumed to reduce their overall clinical treatment benefit.

Our study demonstrates that the majority of patients with Stage III/IV NSCLC in routine clinical practice can maintain or improve physician-rated PS (KI) and patient-rated HR-QoL (EQ-5D) during second-line treatment with single-agent pemetrexed. Our population included 36% of elderly patients. The majority of all patients (58.0%) achieved KI benefit response after the second treatment cycle (with 90% of patients still on study), and KI benefit response rates increased further during the subsequent cycles. Without second-line treatment, PS and HR-QoL would most likely have declined due to the disease progression. Even with treatment, a decline in PS and HR-QoL would be expected for patients with disease progression or major toxicities. Correspondingly, patients who had experienced no Grade 3/4 toxicities during the first 2 cycles had a significant advantage with respect to achieving KI benefit response. Patients with high baseline KI also had a significant advantage with respect to achieving KI benefit response, indicating that it is important to optimize patients' general condition before starting second-line treatment.

Several studies have underlined the importance of considering PS for clinical practice. Lilenbaum et al. found high prevalences of poor PS (Eastern Cooperative Oncology Group [ECOG] PS 2-4, corresponding to a KI < 80%) in lung cancer patients of 34% when rated by physicians and of 48% when rated by patients [10]. Blagden et al. showed that both patient-and physician-rated PS scores reflected duration of survival and disease stage; physician-rated scores were only marginally more predictive of survival [8]. A recent study in more than 26,000 NSCLC patients has shown that PS is an independent risk factor for patient OS [11].

No previous study has looked at changes in PS during second-line treatment. Only one post-hoc analysis of data from a first-line treatment study has been published by Sculier et al. [27]. In their study, 485 patients received three cycles of triple-agent treatment with cisplatin, gemcitabine and ifosfamide. 25% of patients who had a poor baseline KI of 60% to 70% achieved clinical improvement, defined as high KI of ≥ 80%. These findings are consistent with the improvement rate of 20.8% in patients with poor baseline KI we observed with second-line pemetrexed treatment.

We additionally looked at patient-rated HR-QoL, using the EQ-5D self-estimation instrument. The EQ-5D was chosen because it is a simple, validated and commonly used questionnaire which can be completed within a short time [15]. The disease-specific, multidimensional tools commonly used in lung cancer studies, such as the Lung Cancer Symptom Scale (LCSS) used by Hanna et al. [6] in a study which compared second-line treatment with pemetrexed versus docetaxel, were too complex for use in a routine clinical practice setting. The EQ-5D has been validated into 36 official languages (http://www.euroqol.org), including the German language [28]. We found that on average, baseline EQ-5D ratings were at least maintained, consistent with the physician-based KI ratings. Mean EQ-5D index and EQ-VAS scores even showed small improvements after the second treatment cycle, i.e., as early as 6-weeks after the initiation of pemetrexed treatment. The EQ-5D instrument has rarely been used in NSCLC patients so far. Grutters et al. have recently applied the EQ-5D in patients surviving lung cancer. They found that patients with severe adverse events (dyspnoea grade ≥ 3) during treatment had statistically significantly lower EQ-5D index scores than patients without severe adverse events [29], indicating that the instrument is sensitive enough to detect health status changes during chemotherapy.

In our study, second-line pemetrexed treatment was well tolerated by most patients. Two of 516 patients (0.4%) died due to drug-related toxicity. There were no signs of cumulative toxicities. On the contrary, Grade 3/4 toxicity rates decreased after repeated treatment cycles. Patients in our study received a higher number of treatment cycles than in the randomized phase III trial of second-line pemetrexed treatment by Hanna et al. [6] which allowed for treatment up to disease progression (median 5 vs. 4 cycles). In our study, 20% of patients received at least 9 cycles and 5% continued pemetrexed treatment after the end of study. Considering in addition that most patients maintained or improved their PS and HR-QoL, our data indicate that a high number of treatment cycles do not impair patients' quality of life.

The Grade 3/4 toxicity profile, recorded by solicited questioning of specific non-hematologic toxicities, was largely comparable to the unsolicited toxicity rates during pemetrexed treatment published by Hanna et al. (e.g. febrile neutropenia 1.9% vs. 1.9%, nausea 3.1% vs. 2.6%, vomiting 1.5% vs. 1.0%, rash 1.4% vs. 0.8%). However, we found higher rates of Grade 3/4 asthenia/fatigue (15.9% vs. 5.3%) and Grade 3/4 neutropenia (8.7% vs. 5.3%).

Further, we found no evidence that Grade 3/4 toxicity rates were higher in elderly than in younger patients. These findings are well in line with a subgroup analysis of toxicities in the Hanna study, performed by Weiss et al., which also did not find any significant difference in toxicities between older and younger patients ( ≥ 70 vs. < 70 years) [30]. A previous metaanalysis of 3 studies on pemetrexed first-line treatment in 764 NSCLC patients also concluded that pemetrexed or pemetrexed-based combinations produced similar treatment effects in older and younger patients ( ≥ 65 and < 65 years) and appeared to be well tolerated in the elderly population [31]. Thus, it cannot be stated in general that treatment toxicity is higher in elderly patients; this may depend on the type of treatment used. A recent retrospective study by Chrischilles et al. evaluated chemotherapy use and adverse events during treatment of advanced NSCLC in routine clinical practice and concluded that toxicity was increased in elderly patients, but his study mainly looked at first-line treatment with platinum-based combinations [32]. One additional result of this US study was that physicians used carboplatin-based combinations more frequently than cisplatin-based combinations (65.3% vs. 10.4%). Our German and Austrian data were fully consistent with these findings: Physicians used carboplatin-based combinations as first-line treatment in approximately two thirds of patients (carboplatin 62.8%, cisplatin 25.9%). Administration of carboplatin is more convenient and less emetogenic than cisplatin [33], and carboplatin has been associated with less toxicity [34]. This may explain why the latter is preferred in routine clinical practice.

Our study has limitations. First, the non-interventional design to observe patients in routine clinical practice prohibits any definite conclusion on the efficacy of second-line pemetrexed treatment. In particular, tumor response data are of limited value and cannot be compared to clinical trial data because physicians may not have performed standard radiologic assessments or classified response in accordance with the standard response evaluation criteria in solid tumors (RECIST). Second, our toxicity data cannot be directly compared with toxicity data from clinical trials, because the collection of toxicities differed substantially: only a few solicited toxicities were specifically asked for via tick-boxes; no complete lists of Common Terminology Criteria for Adverse Events (CTC-AE) were handed out to the investigators. Patients may also have reported e.g. fatigue more frequently due to the solicited questioning for specific toxicities. The validity of these data may therefore be questioned, although the majority of toxicity results were in line with previous clinical studies of pemetrexed [6]. Further, we did not differentiate between in-and outpatient treatment although it can be assumed that the majority of patients received the 10-minute infusion either in outpatient practice or in the ambulance of the hospital. Finally, only half of the patients returned completed EQ-5D questionnaires. The internal validity of our QoL data may therefore be compromised because the missing data may be informative and not at random. The major strength of our study is that we looked at second-line treatment in a large sample of patients as routinely treated in clinical practice where there is a particular lack of data [6]. Clinical trials in NSCLC patients are often criticized for rarely enrolling elderly patients or patients with poor PS. In the randomized phase III trial by Hanna et al., 15.1% of patients in the pemetrexed arm were ≥ 70 years old, and 10.6% of patients had a poor baseline ECOG-PS of 2 [6,30]. In our study, 36.1% of patients were ≥ 70 years of age, and 37.8% of patients had a poor baseline KI of < 80%. Despite the higher proportions of elderly and poor-performance patients in our study, overall disease control rates with pemetrexed were comparable to those found by Hanna et al. (60.3% vs. 54.9%).

## Conclusions

In this large non-interventional study of second-line pemetrexed treatment in patients with Stage III/IV NSCLC, including 36% elderly ( ≥ 70 years) patients, physician-rated PS (KI) and patient-rated HR-QoL (EQ-5D) were maintained or improved in the majority of patients. Both, physician-rated PS and patient-rated HR-QoL assessments should, perhaps, be given more weight than minor radiologic changes in guiding treatment decisions and evaluating the effectiveness of second-line therapy. Patients with high baseline PS also had a significant advantage, showing that it is important to optimize the patients' general condition before starting treatment. Treatment was well-tolerated by the majority of patients and there were no signs of cumulative toxicities. Patients received a higher number of treatment cycles than in clinical trials of second-line pemetrexed treatment; considering that most patients maintained or improved their PS and HR-QoL, our data indicate that a high number of treatment cycles do not impair patients' quality of life. Grade 3/4 toxicity rates were higher in patients with poor baseline PS, but not in elderly versus younger patients, indicating that elderly patients can be considered for second-line treatment with pemetrexed if they have a high PS of ≥ 80.

## Competing interests

WS has served as consultant for and has received research grants from Lilly Deutschland GmbH, Bad Homburg, Germany. HT has served as consultant for and has received honoraria from Sanofi Aventis, Roche Pharma AG, Norvartis, Glaxo Smith Kline, Pfizer and Hexal. HB, TK, VS and CS are employees of Lilly Deutschland GmbH, Bad Homburg, Germany, HB and TK own Eli Lilly stocks.

## Authors' contributions

CS and HB participated in the design of the study. HB and TK were involved in the conduct of the study. VS was responsible for the statistical analysis and drafted the manuscript supported by a medical writer. All authors contributed to data interpretation and revised the draft critically for scientific content. All authors read and approved the final version of the manuscript.

## Pre-publication history

The pre-publication history for this paper can be accessed here:

http://www.biomedcentral.com/1471-2407/12/14/prepub
